# The coalescent with replication-independent mutations

**DOI:** 10.7717/peerj.12926

**Published:** 2022-02-15

**Authors:** Stephen M. Krone, Beth M. Tuschhoff

**Affiliations:** Department of Mathematics and Statistical Science, University of Idaho, Moscow, Idaho, United States

**Keywords:** Coalescent, Replication-independent mutations, Replication rate, Microbial diversity

## Abstract

We develop the mathematical structure of the neutral coalescent with both replication-dependent and replication-independent mutations. This allows us to explain and quantify empirical results that explore differences in genetic diversity in bacterial cultures with different growth rates. We also derive an unbiased and consistent estimator for the replication-independent mutation rate that is based on a comparison of total single nucleotide polymorphism counts for two independent well-mixed cultures with different growth rates. In addition to explaining differences in genetic diversity between well-mixed cultures with different (but constant) growth rates, our coalescent also quantifies the effects of fluctuating growth rates—a situation that can be common in natural populations.

## Introduction

There are two types of mutations: *replication-dependent* mutations occur during reproduction events, and *replication-independent* mutations arise independently of reproduction. This is seen most clearly in empirical work on bacteria in growth-limited conditions (Ryan, 1955; Kivisaar, 2010; France, Ridenhour & Forney, 2018). Replication-independent mutations arise in a variety of ways, including error-prone repair of DNA that has become damaged through UV radiation, oxidation, or alkylation (France, Ridenhour & Forney, 2018; Kivisaar, 2010). Such mutations are sometimes attributed to a stress response, though that need not be the case. Microbial populations can have growth rates that vary widely—with doubling times ranging from a fraction of an hour in lab cultures to days, weeks, or longer in environmental settings (Morita, 1988; France, Ridenhour & Forney, 2018). This suggests that the role of replication-independent mutations in the generation of microbial diversity should also vary widely. In this paper, we derive the mathematical structure of the coalescent describing the genealogy of a sample for a version of the neutral Wright–Fisher model that includes both replication-dependent and replication-independent mutations. We ignore selective eﬀects in the generation and fixation of mutants, focusing on the accumulation of neutral genetic diversity. This diversity, of course, can provide standing genetic variation that contains the seeds of adaptive evolution once selective pressure is applied, say through the introduction of an antibiotic (France, Ridenhour & Forney, 2018).

Our interest in this topic was inspired by empirical work of France (2018), France, Ridenhour & Forney (2018) and France et al. (2019) that demonstrated the eﬀects of reduced growth rates on the presence of mutations in bacterial populations that were grown in chemostats and biofilms. In particular, they compared two chemostat cultures with the same population density, one with a ‘slow’ growth rate and the other with a ‘fast’ growth rate. They found enhanced bacterial diversity in the slowly growing culture compared to faster growing culture when the populations were compared after the same number of generations. When comparisons were made at the same amount of ‘real time,’ the faster-growing culture displayed the larger number of mutations. Coalescent theory, which is based on sampling existing genetic diversity in the present based on the shared ancestry of individuals in the sample, is a generation-based construct. As such, a coalescent that accounts for replication-independent mutations should predict more genetic diversity in slowly growing populations.

Blath et al. (2015) studied a related situation involving spore-forming cells in which a given microbial population is divided into subpopulations, with one containing actively growing cells and the other dormant cells. They model this with a neutral seed bank structure and derive a coalescent describing the ancestry of a sample. Our approach contrasts with the one in Blath et al. (2015) in that we deal with populations that (1) experience periods of reduced growth rather than complete dormancy and (2) are not spatially or otherwise structured in a way that keeps individuals in the reproducing or non-reproducing subpopulations over an extended period. For example, our model corresponds to bacteria growing in a chemostat in which nutrient levels can be changed to alter growth rates; the models of Blath et al. (2015) are more appropriate for bacteria growing in a biofilm, where parts of the population do not have access to nutrients and do not grow, and others are actively growing. Although Blath et al. (2015) have replication-independent mutations in their model, their analyses focus on cases where these mutations are ignored. We find that replication-independent mutations are important in explaining certain empirical results, and so we emphasize their role in our analyses.

In theoretical population genetics, forward-time mathematical models typically include mutation as a component of generational events (reproduction). The standard approach in diﬀusion theory and coalescent theory is to assume a Wright–Fisher model (Wakeley, 2009) with mutation probability per individual per reproduction of the form *u* = *θ*/(2*N*), where *N* is population size (assumed constant in the simplest cases) and *θ* is the scaled (replication-dependent) mutation rate. With this scaling and a speed up of time (*t* units of continuous time corresponding to ⌊*Nt*⌋ generations), one obtains a Wright–Fisher diﬀusion in forward time and Kingman’s coalescent in reverse time. This theme has been expanded upon to include the eﬀects of more complex dynamics by incorporating additional structure in the Wright–Fisher dynamics. Two examples of such additional structure are pertinent to the present study. In one example, Kaj & Krone (2003) and Sjödin et al. (2005) investigated the structure of the coalescent in the presence of stochastically fluctuating population sizes. The appropriately scaled ancestral process converges to a coalescent with constant mutation rate *θ*/2 along each lineage and pairwise coalescence rates that change with time. An equivalent rescaling (in terms of predicting the same mutation frequencies in a sample) of this coalescent has constant pairwise coalescence rates but mutation rates that vary with time. A second example is the seed bank coalescent introduced by Kaj, Krone & Lascoux (2001). In this case, not all oﬀspring come from parents in the previous generation, with some “seeds” being sequestered for a time before they result in progeny. This sequestration of part of the population results in a slowing of coalescence rates and a consequent increase in diversity. Blath et al. (2015) expanded on this by allowing for a more general “strong seed bank” structure, and they were the first to propose its use to model microbial diversity.

## Model development and coalescent structure

We derive the mathematical structure of the coalescent when both replication-independent and replication-dependent mutations are present. We will see how the two types of mutations operate on diﬀerent time scales—a result that can be put to use in estimating rates of replication-independent mutations and explaining recent empirical data on microbial diversity. Reproduction (or replication) events involve replication of the genome of the reproducing “parent,” and this process is subject to replication errors, leading to replication-dependent mutations. Replication-independent mutations arise over time independently of replication. In order to account for both sources of mutation in our model, we must be able to partially decouple time steps and replication events.

*Reproduction:* Our forward-time model is an extension of the discrete-time haploid Wright–Fisher model with fixed population size *N* in which, instead of replacing all *N* individuals with their oﬀspring each time step, only a fraction of them (randomly chosen) is replaced—resulting in a decoupling of time steps and “generations,” as will be discussed later. This replacement fraction is assumed to depend on growth conditions (something easily controlled in a lab, for example, by regulating nutrient levels and dilution rate for bacteria in a chemostat). Since we are interested in a retrospective approach, we denote by *R*_*N*_(*k*) the number of individuals in the population who are replaced *k* time steps in the past; thus 0 ≤ *R*_*N*_(*k*) ≤ *N*. The *N* individuals in the population alive *k* time steps in the past thus arose from the previous time step through *R*_*N*_(*k*) replacements and *N* − *R*_*N*_(*k*) individuals surviving to the next time step. Thus, the replicating part of the population undergoes the usual Wright–Fisher dynamics with all *R*_*N*_(*k*) parents dying and being replaced with *R*_*N*_(*k*) oﬀspring. The special case *R*_*N*_(*k*) ≡ *N* yields the usual Wright–Fisher model. Two specific individuals in time step *k* − 1 will have come from a reproduction event from the same parent in time step *k* if they both were the products of reproducing parents and, given this, they had the same parent. Thus

$$\eqalign{
  & P({\rm{a}}\;{\rm{given}}\;{\rm{pair}}\;{\rm{in}}\;{\rm{time}}\;{\rm{step}}\;k - 1\;{\rm{coalesces}}\;{\rm{to}}\;{\rm{a}}\;{\rm{common}}\;{\rm{parent}}\;{\rm{in}}\;{\rm{time}}\;{\rm{step}}\;k) =   \cr 
  & \;{\left( {{{{R_N}(k)} \over N}} \right)^2} \cdot {1 \over {{R_N}(k)}} = {{{R_N}(k)} \over {{N^2}}}. \cr} $$

Note that the intermediate step in this equation does not work in the special case *R*_*N*_(*k*) = 0, but the final result holds trivially since there can be no coalescing in a time step where there was no replication. The special case of Wright–Fisher dynamics, *R*_*N*_(*k*) = *N*, yields the usual 1/*N* pairwise coalescence probability.

To construct the coalescent process, assume that the scaled ‘replacement’ process converges to a *replication rate* process *ψ*(*t*) ∈ [0, 1]:

(1) $${{{R_N}(\left\lfloor {Nt} \right\rfloor )} \over N} \to {\rm{ }}\psi (t)$$

as $N \to \infty$. Roughly speaking, this means that the average number replaced per time step is of order *N*; *e.g*., *R*_*N*_(*k*) = *c*_*k*_*N*, where *c*_*k*_ ∈ [0, 1]. The convergence in Eq. (1) is weak convergence of stochastic processes and includes as special cases of *ψ*(*t*) continuous-time Markov chains, diﬀusion processes, and deterministic functions of time. See (Kaj & Krone, 2003; Sjödin et al., 2005) for examples and the technical details of convergence in a model with stochastically fluctuating population sizes, which has some features similar to those in the model here.

With the above convergence, the probability that two specific lineages do not coalesce in ⌊*Nt*⌋ time steps, given the sequence of *R*_*N*_(*k*) values, is

$$\eqalign{
  & {P_2}({\rm{no}}\;{\rm{coalescence}}\;{\rm{in }}\left\lfloor {Nt} \right\rfloor {\rm{ time}}\;{\rm{steps}}\;|{R_N}( \cdot )) = \prod\limits_{k = 1}^{\left\lfloor {Nt} \right\rfloor } {\left( {1 - {{{R_N}(k)} \over {{N^2}}}} \right)}   \cr 
  & \quad \quad \quad \quad \quad \quad \quad \quad \quad \quad \quad \quad \quad \quad \quad \quad \quad \quad \~{\mathop{\rm exp}\nolimits} \left( { - {1 \over N}\sum\limits_{k = 1}^{\left\lfloor {Nt} \right\rfloor } {{{{R_N}(k)} \over N}} } \right)  \cr 
  & \quad \quad \quad \quad \quad \quad \quad \quad \quad \quad \quad \quad \quad \quad \quad \quad \quad \quad  \to {\mathop{\rm exp}\nolimits} \left( { - \int_0^t \psi  (s)ds} \right), \cr} $$

as $N \to \infty$. Thus, on the coalescent time scale, *ψ*(*t*) is the pairwise coalescence rate at time *t* in the past. This agrees with the standard pairwise coalescence rate of 1 when we have full replacement each generation.

A simple example of the framework indicated by Eq. (1) has *R*_*N*_(*k*) taking values in {0, *N*}, so that we get full replacement in some time steps and no replacement in others. If the transition probabilities for the process (*R*_*N*_(*k*)) are of the form *p*(0, *N*) = *λ*_1_/*N* and *p*(*N*, 0) = *λ*_0_/*N*, then the replication rate process (*ψ*(*t*)) takes values in {0, 1} and has transition rates *q*(0, 1) = *λ*_1_ and *q*(1, 0) = *λ*_0_. In this situation, coalescence events and replication-dependent mutations would arise according to a standard coalescent while *ψ*(*t*) = 1 and they would pause while *ψ*(*t*) = 0. Replication-independent mutations would arise independently of the value of *ψ*(*t*).

We remark briefly that, in the above model description, *R*_*N*_(*k*) ≡ 1 is formally equivalent to the Moran model (Wakeley, 2009), though this model assumes that all parents are equally likely to serve as the lone parent in a given time step, and hence the coalescence of lineages going back in time requires a slightly diﬀerent interpretation. If we had chosen *R*_*N*_(*k*) to be of order 1, instead of *N*, we would need a diﬀerent scaling of time ([*N*^2^*t*] instead of [*Nt*]) in (1). We will not pursue the Moran scaling in this paper.

*Mutation:* All mutations in our model are assumed to follow the “infinite sites” assumption, with each mutation being distinguishable from all others (Wakeley, 2009). With the usual *replication-dependent mutation* probability per individual *per reproduction* scaled as *u* = *θ*/(2*N*), we obtain a scaled replication-dependent mutation rate ${\theta \over 2} \cdot {\it \psi} (t)$ along each lineage in the limiting coalescent process. This follows by a calculation similar to the one in the above displayed equation but, at time step *k* in the past, the probability that a given lineage experiences a replication-dependent mutation is

$\displaystyle{{{R_N}(k)} \over N} \cdot \displaystyle{\theta \over {2N}}\; ,$

the probability that a specific individual replicates, and the oﬀspring acquires a new replication-dependent mutation.

To account for the diﬀerent patterns of mutations seen in empirical data for growth-limiting cultures, we need another source of mutations that is not tied to reproduction events. We thus further assume that, in the Wright–Fisher construction, there is an additional probability *w* = *ν*/(2*N*) of a *replication-independent mutation* for each individual in the population *per time step*, regardless of their status as a new oﬀspring or a surviving individual from the previous time step. An even simpler calculation of the above form shows that, in the limiting coalescent, this will correspond to a constant rate *ν*/2 of replication-independent mutations along each lineage.

*Remark:* Note that the same scaling of coalescence and replication-dependent mutation obtains. If replication-independent mutations were not present (*ν* = 0), patterns of replication-dependent mutations in such a tree (or in a sample of SNPs from such a population with varying replication rates) would be indistinguishable from those in the standard coalescent. This is not the case, for example, in the coalescent with stochastically varying population size (Kaj & Krone, 2003; Sjödin et al., 2005) or in the seed bank coalescent (Kaj, Krone & Lascoux, 2001). In the present model, it is the additional replication-independent mutations that lead to diﬀerences from the standard coalescent.

Combining the eﬀects of reproduction and the two types of mutation, in the limit as $N \to \infty$, we obtain a coalescent process that can be viewed in two ways:

**I. The coalescent process with replication-dependent and replication-independent mutations**, defined by the rates

- pairwise coalescence rate *ψ*(*t*),
- replication-dependent mutation rate per lineage $\displaystyle{\theta \over 2} \cdot {\it \psi} (t)$,
- replication-independent mutation rate per lineage $\displaystyle{\nu \over 2}$.

These rates correspond to a scaling of coalescence time in units of “time steps” in the revised Wright–Fisher model; *t* units of coalescent time corresponds to ⌊*Nt*⌋ time steps in the WF model.

If we re-scale coalescence rates and mutation rates by the same amount (even if that scaling depends on *t*), the distribution of branch lengths will change but we obtain the same distribution of mutations along the branches. Thus, as far as polymorphism data are concerned, it is equivalent to use.

**II. The re-scaled coalescent with replication-dependent and replication-independent mutations**, defined by the rates

- pairwise coalescence rate 1,
- replication-dependent mutation rate per lineage $\displaystyle{\theta \over 2}$,
- replication-independent mutation rate per lineage $\displaystyle{\nu \over {2{\it \psi} (t)}}$.

In this scaling, of course, we need *ψ*(*t*) > 0 for all *t*. These re-scaled rates correspond to a scaling of coalescence time in units of “generations” (the average time it takes to replace *N* individuals) in the revised Wright–Fisher model; *t* units of coalescent time corresponds to ⌊*Nt*⌋ generations in the WF model. (Note that time steps and generations are no longer the same in our revised Wright–Fisher model; not all individuals are involved in the reproduction-replacement dynamics.)

In other words, we can obtain the re-scaled coalescent process with replication-dependent and replication-independent mutations by generating the standard Kingman coalescent and then adding replication-independent mutations at rate *ν*/(2*ψ*(*t*)) per lineage at time *t* in the past. This version is easier to work with since the replication rate appears only in the “new” replication-independent mutation rate. Note that it also implies that the number of replication-independent mutations increases as *ν* increases or *ψ*(*t*) decreases; only the ratio matters.

## Applications

Two distinct settings are relevant for application of this coalescent:

1. *Constant replication rate*: the replication rate *ψ*(*t*) is constant, but we compare polymorphism data for several diﬀerent values of this constant;
2. *Fluctuating replication rate*: the replication rate *ψ*(*t*) is changing with time quickly enough that we see changes of replication rate in the coalescent.

### Comparing populations with different constant replication rates

In all that follows, we use abbreviations RD and RI for replication-dependent and replication-independent mutations. We begin by considering how RI mutations and replication rate combine to aﬀect the number of segregating sites in a sample from a single population with constant replication rate. A constant replication rate, *ψ*(*t*) = *c* ∈ (0, 1], will yield a “generation” (defined as the time it takes for replacement of *N* individuals in a population of size *N*) every 1/*c* time steps on average. We will exploit this diﬀerence between time steps and generations to provide a method for determining the rate of replication-independent mutations, even though we cannot directly distinguish these from replication-dependent mutations in practice.

Recall first some basic results from coalescent theory (Wakeley, 2009). Writing *T*_*i*_ for the time (in the coalescent) until the next coalescence when there are *i* lineages, and ${L_n} = \sum_{i = 2}^n i {T_i}$ the total branch length in the tree for a sample of size *n*, we have *E*(*L*_*n*_) = 2*a*_*n*_ and Var(*L*_*n*_) = 4*b*_*n*_, where ${a_n} = \sum_{i = 1}^{n - 1} 1 /i$ and ${b_n} = \sum_{i = 1}^{n - 1} 1 /{i^2}$. Assume the infinite sites model of mutation and let $S_n^{(RD)}$ denote the number of single nucleotide polymorphisms (SNP) in a sample of size *n* that were generated by RD mutations. We have $E(S_n^{(RD)}) = \theta {a_n}$ and ${\rm Var}(S_n^{(RD)}) = \theta {a_n} + {\theta ^2}{b_n}$, where *θ*/2 is the per-lineage RD mutation rate.

The quantities *T*_*i*_ and *L*_*n*_ in the coalescent correspond to *NT*_*i*_ and *NL*_*n*_ generations when the population size is fixed at *N* and all *N* individuals are replaced by oﬀspring each generation. Now, if the replication rate is fixed at *ψ*(*t*) = *c* ∈ (0, 1), the rates of coalescence and RD mutation both slow by a factor of *c* compared to a standard coalescent with replication rate 1, so $S_n^{(RD)}$ has the same distribution, independent of *c* > 0. Since we are interested in the pattern of SNPs in a sample, only the relative rates matter and we can use the re-scaled coalescent process (II) when considering the added eﬀects of RI mutations. In particular, the mean and variance of $S_n^{(RD)}$ do not depend on *c*. If, however, we let $S_n^{(RI,c)}$ denote the number of RI mutations in a sample of size *n*, this quantity depends on actual time instead of generations, and so is a function of the replication rate *c*. In particular, the total branch length *L*_*n*_, in generations, corresponds to *L*_*n*_/*c* in real time units. So the eﬀective rate of RI mutations on the coalescent scale is *ν*/2*c*. Hence $E(S_n^{(RI,c)}) = E[E(S_n^{(RI,c)}|{L_n})] =\displaystyle{\nu \over {2c}} \cdot 2{a_n} = \displaystyle{\nu \over c} \cdot {a_n}$ and, similarly, ${\rm Var}(S_n^{(RI,c)}) = E[{\rm Var}(S_n^{(RI,c)}|{L_n})] + {\rm Var}[E(S_n^{(RI,c)}|{L_n})] = \displaystyle{\nu \over c} \cdot {a_n} + \displaystyle{{{\nu ^2}} \over {{c^2}}} \cdot {b_n}$.

Combining the two types of mutations, the *total number of SNPs* in a sample of size *n* is

$S_n^{(c)} = S_n^{(RD)} + S_n^{(RI,c)}.$

When the replication rate is fixed at *c*,

(2) $$E(S_n^{(c)}) = {a_n}\left( {\theta + \displaystyle{\nu \over c}} \right)$$

and

(3) $$\eqalign{
  & {\rm{Var}}(S_n^{(c)}) = {\rm{Var}}(S_n^{(RD)}) + {\rm{Var}}(S_n^{(RI,c)}) + 2{\rm{Cov}}\left( {S_n^{(RD)},S_n^{(RI,c)}} \right)  \cr 
  & \quad \quad \quad \quad  = {a_n}\left( {\theta  + {\nu  \over c}} \right) + {b_n}\left( {{\theta ^2} + {{{\nu ^2}} \over {{c^2}}}} \right) + {{2\theta \nu } \over c}{b_n}  \cr 
  & \quad \quad \quad \quad  = {a_n}\left( {\theta  + {\nu  \over c}} \right) + {b_n}{\left( {\theta  + {\nu  \over c}} \right)^2}. \cr} $$

Of course, these results follow even more directly by recognizing that the rescaled version of our coalescent (II), in the special case where *ψ*(*t*) = *c*, is equivalent to the standard coalescent with (total) mutation rate $(\theta + {\nu \over c})/2$. Similarly, it follows (Wakeley, 2009) that the average number of pairwise diﬀerences, $\pi _n^{(c)}$, in a sample of size *n* satisfies

(4) $$E(\pi _n^{(c)}) = \theta + \displaystyle{\nu \over c}$$

and

(5) $${\rm Var}(\pi _n^{(c)}) = \displaystyle{{n + 1} \over {3(n - 1)}}\left( {\theta + \displaystyle{\nu \over c}} \right) + \displaystyle{{2({n^2} + n + 3)} \over {9n(n - 1)}}{\left( {\theta + \displaystyle{\nu \over c}} \right)^2}$$

and the *i*th element in the site frequency spectrum has mean

(6) $$E({\xi _i}) = (\theta + \displaystyle{\nu \over c})/i.$$

**Figure 1:**
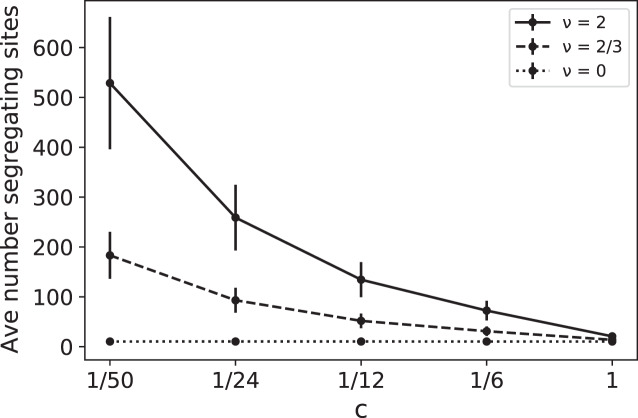
Average number of segregating sites, $S_{100}^{(c)}$, for a sample of size *n* = 100 as a function of replication rate *c* for three diﬀerent values of RI mutation rate *ν*. The RD mutation rate is *θ* = 2 and each data point corresponds to 100,000 runs. For *ν* = 0, the constant value of the sample means for $S_{100}^{(c)}$ was approximately 10.35 with sample standard deviation *σ* ≈ 4.1. The predicted expected value for the case *ν* = 0 is $E(S_{100}^{(c)}) = {a_{100}}\theta \approx 5.177 \times 2 \approx 10.35$ RD mutations for each value of c, clearly within one standard deviation of the mean. The other simulated means in the graph (corresponding to *ν* = 2/3 and 2) were also found to be within one standard deviation of the theoretical mean $E(S_{100}^{(c)}) = {a_{100}}(\theta + \nu /c)$.

Note that the eﬀects of *ν* and *c* are intertwined, always appearing together in the ratio *ν*/*c*. For example, doubling the RI mutation rate has the same eﬀect as reducing the replication rate by 1/2. In Fig. 1 we plot simulated values of $S_n^{(c)}$ as a function of *c* for several diﬀerent values of the RI mutation rate *ν*. These simulations provide a coalescent-based rendering of the types of data found in France (2018). In Fig. 1, for a given *ν* > 0, the increase in $S_n^{(c)}$ as *c* decreases is due to the fact that slower replication provides more opportunity for RI mutations to accumulate.

**Estimating the RI mutation rate.** Consider now the case where we are able to compare SNP data from two independent cultures of the same species, with constant replication rates: one slow with rate *c*_1_ and one fast with rate *c*_2_ (0 < *c*_1_ < *c*_2_). Since one expects the same number of replication-dependent mutations in the two cultures, the average diﬀerence between total numbers of mutations, $S_n^{({c_1})} - S_n^{({c_2})}$, will be accounted for by the rate of replication-independent mutations. Indeed,

(7) $$E\left( {S_n^{({c_1})} - S_n^{({c_2})}} \right) = {a_n}\nu \left( {\displaystyle{1 \over {{c_1}}} - \displaystyle{1 \over {{c_2}}}} \right),$$

which leads to an unbiased *estimator of the RI mutation rate*:

(8) $$\hat \nu = \displaystyle{{S_n^{({c_1})} - S_n^{({c_2})}} \over {{a_n}\left( {\displaystyle{1 \over {{c_1}}} - \displaystyle{1 \over {{c_2}}}} \right)}}\; .$$

Thus $E(\hat \nu ) = \nu$ and, since $S_n^{({c_1})}$ and $S_n^{({c_2})}$ are independent,

(9) $$\eqalign{
  & {\rm{Var}}(\hat{\nu }) = {{{\rm{Var}}\left( {S_n^{({c_1})} - S_n^{({c_2})}} \right)} \over {a_n^2{{\left( {{1 \over {{c_1}}} - {1 \over {{c_2}}}} \right)}^2}}} = {1 \over {a_n^2{{({1 \over {{c_1}}} - {1 \over {{c_2}}})}^2}}}[{a_n}(2\theta  + \nu ({1 \over {{c_1}}} + {1 \over {{c_2}}}))  \cr 
  & \quad \quad  + {b_n}(2{\theta ^2} + {\nu ^2}({1 \over {c_1^2}} + {1 \over {c_2^2}})) + 2\theta \nu {b_n}({1 \over {{c_1}}} + {1 \over {{c_2}}})]. \cr} $$

**Figure 2:**
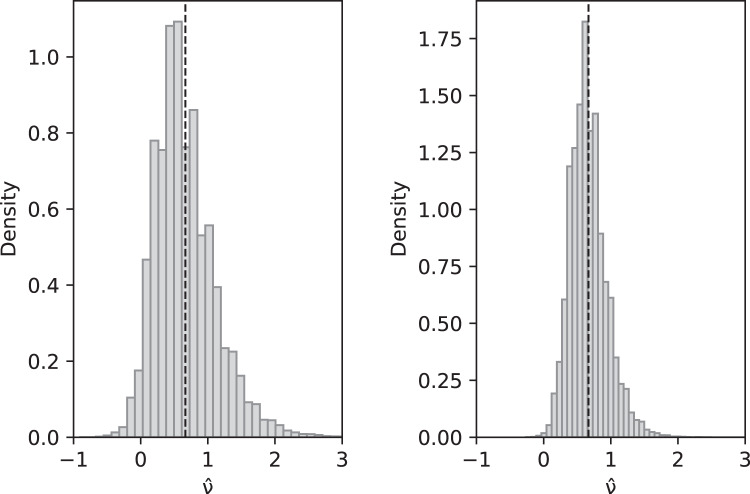
Simulated values of $\hat \nu$ with sample size *n* = 10 (left) and *n* = 100 (right) when actual value is *ν* = 2/3 and parameters are *θ* = 2, *c*_1_ = 1/12 (for slow-growth population), *c*_2_ = 1 (for fast-growth population). Left: sample mean 0.6648, sample variance 0.2060; Right: sample mean 0.6677, sample variance 0.0744. These are a close match to the theoretical values $E(\hat \nu ) = 2/3$ and ${\rm Var}(\hat \nu ) = 0.2073$ for *n* = 10 and 0.0742 for *n* = 100.

Asymptotically, since *a*_*n*_ ∼ ln(*n*) and *b*_*n*_ ∼ *π*^2^/6, ${\rm Var}(\hat \nu ) \to 0$ as $n \to \infty$, and hence $\hat \nu$ is also a consistent estimator of *ν*. Compare with Fig. 2.

### Fluctuating replication rates

In the case of fast fluctuations in replication rate, we can compute the *coalescent eﬀective population size* as in Kaj & Krone (2003) and Sjödin et al. (2005). Intuitively, if the growth rate changes fast enough, it will reach equilibrium between coalescence and mutation events. If *ψ*(*t*) has unique stationary distribution *π*, then the resulting ancestral dynamics will be equivalent to that of a population with constant growth rate $c = \int_{[0,1]} x {\mkern 1mu} \pi (dx)$ and the coalescent eﬀective size will be *N*_*e*_ = *N*/*c*. The process thus behaves as though there was a constant growth rate and an overall eﬀective mutation rate *c* ⋅ *θ*/2 + *ν*/2 per lineage.

**Figure 3:**
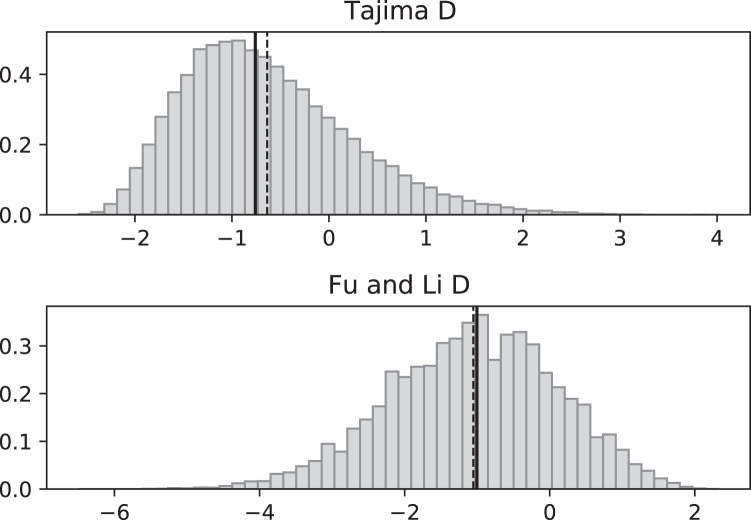
Fluctuating replication rates with RI mutation rate *ν* = 2/3 and slow initial replication. Simulated values of Tajima and Fu and Li D statistics for fluctuating replication rates *ψ*(*t*) switching between *c*_1_ = 1/12 and *c*_2_ = 1 at rate 2 (starting with slower replication rate *c*_1_). Simulations used *n* = 100, *θ* = 2, *ν* = 2/3, and 100,000 replicates. The means (dashed vertical lines) are *D*_*T*_ = −0.6369 and *D*_*FL*_ = −1.0539. The medians (solid vertical lines) are *D*_*T*_ = −0.7593 and *D*_*FL*_ = −1.0027.

**Figure 4:**
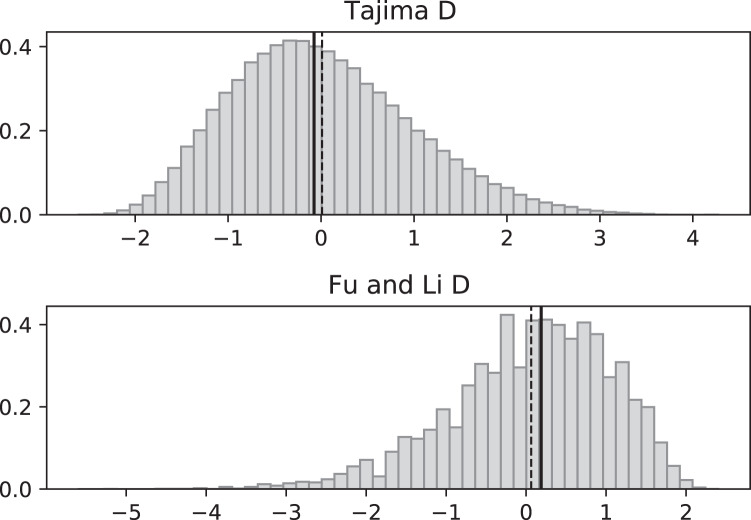
Fluctuating replication rates with RI mutation rate *ν* = 2/3 and fast initial replication. Simulated values of Tajima and Fu and Li D statistics for fluctuating replication rates *ψ*(*t*) switching between *c*_1_ = 1/12 and *c*_2_ = 1 at rate 2, as in Fig. 3, but this time starting with the faster replication rate *c*_2_. Simulations used *n* = 100, *θ* = 2, *ν* = 2/3, and 100,000 replicates. The means (dashed vertical lines) are *D*_*T*_ = 0.0111 and *D*_*FL*_ = 0.0640. The medians (solid vertical lines) are *D*_*T*_ = −0.0746 and *D*_*FL*_ = 0.1889.

**Figure 5:**
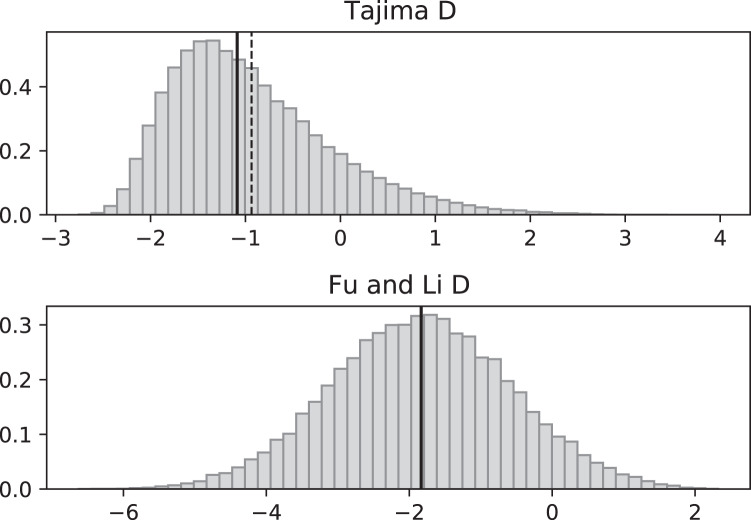
Fluctuating replication rates with RI mutation rate *ν* = 2 and slow initial replication. Increasing the RI mutation rate enhances the eﬀects of fluctuating replication rates. Simulations used *n* = 100, *θ* = 2, *ν* = 2, *ψ* switches between 1/12 and 1 at rate 2 (starting with 1/12), and 100,000 replicates. The means (dashed vertical lines) are *D*_*T*_ = −0.9360 and *D*_*FL*_ = −1.8354. The medians (solid vertical lines) are *D*_*T*_ = −1.0873 and *D*_*FL*_ = −1.8331.

**Figure 6:**
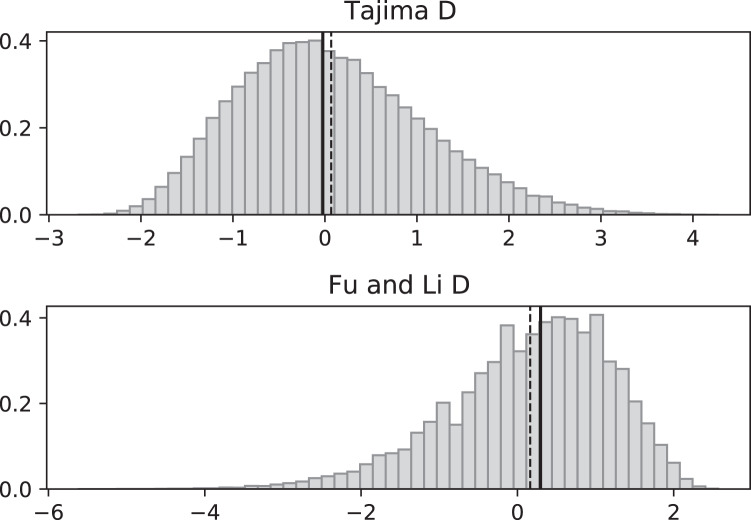
Fluctuating replication rates with RI mutation rate *ν* = 2 and fast initial replication. Simulations used *n* = 100, *θ* = 2, *ν* = 2, *ψ* switches between 1/12 and 1 at rate 2 (starting with 1), and 100,000 replicates. The means (dashed vertical lines) are *D*_*T*_ = 0.0680 and *D*_*FL*_ = 0.1636. The medians (solid vertical lines) are *D*_*T*_ = −0.0233 and *D*_*FL*_ = 0.2965.

In the case of growth rates that fluctuate on the same time scale as coalescence events, there will be no coalescent eﬀective size and we expect our coalescent to generate polymorphism data that deviate from what one expects in a standard, constant population size, neutral coalescent. In Figs. 3–6, we compare the eﬀects of fluctuating replication rates as measured by Tajima’s D (*D*_*T*_) and Fu and Li’s D (*D*_*FL*_) statistics. We use the same versions of *D*_*T*_ and *D*_*FL*_ that are given in Blath et al. (2015). In particular, SNP data that come from a model consistent with the standard Kingman coalescent (in particular, with constant replication rates) should have a mean near 0.

Simulated SNP data were produced from simulations of the coalescent with RI and RD mutations. Deviations from the SNP patterns that would be found in a standard constant-replication-rate coalescent are evident in negative mean and median values of *D*_*T*_ and *D*_*FL*_ when the coalescent begins (at the time of the sample) with the slower rate (Fig. 3). By contrast, starting at the faster replication rate masks many of the eﬀects of RI mutations since the tree experiences more coalescing of lineages before the replication rate fluctuations begin, as seen in mean *D*_*T*_ and *D*_*FL*_ values near 0 (Fig. 4). Thus, sampling from a population that is in a slow-growth phase at the time of the sample is more likely to exhibit diﬀerences from standard SNP patterns. This eﬀect is enhanced when the slow-growth phase is even slower. For example, under the conditions of Fig. 3, if we change *c*_1_ to 1/50, the means become *D*_*T*_ = −0.9521 (instead of −0.6369) and *D*_*FL*_ = −2.2670 (instead of −1.0539). Because of its emphasis of mutations along external branches, *D*_*FL*_ is more sensitive to these patterns than *D*_*T*_.

Figures 5 and 6 repeat the fluctuating size experiments of Figs. 3 and 4, but have a higher RI mutation rate (*ν* = 2 instead of *ν* = 2/3). As expected, the deviations from constant replication rate expectations are enhanced when the RI mutation rate is larger.

## Discussion

Diversity is purged by directional selection and drift—both requiring reproduction. When growth rates are very low, these forces play a minor role relative to replication-independent mutations, allowing diversity to accumulate despite a paucity in the replication-dependent mutations that most models focus on.

It is through the partial decoupling of growth and generation (with a fraction of the population being replaced by oﬀspring in each time step) that we are able to use discrete time steps as a proxy for real time in the implementation of the replication-independent mutation process. In the Wright–Fisher model with population size *N*, one time step is the same as one generation; all *N* individuals are replaced every generation and, retrospectively, it takes an average of *N* generations for a pair of lineages to coalesce. As a simple comparison, consider our revised Wright–Fisher model with *R*_*N*_(*k*) = *N*/3 individuals replaced each time step (*i.e*., the case *c* = 1/3). It takes three time steps to replace *N* individuals in this model, and it takes an average of 3*N* time steps for two lineages to coalesce. This matches the Wright–Fisher model if we now consider one “generation” to be three time steps. The RI mutations accumulate on the basis of time steps, not generations, and hence we expect more of these mutations in the case where the replication rate is slower.

In the limiting coalescent process, one can think of the accumulation of replication-independent mutations as providing a real-time molecular clock (Kumar & Subramanian, 2002). In the absence of replication-independent mutations (*ν* = 0), there is no notion of real time in the coalescent, just generational time. This is seen from the fact that coalescence and replication-dependent mutation rates both scale identically with *ψ*(*t*). In other words, with *ν* = 0, there is no way to discern diﬀerences in growth rates over time or across spatial locations (or diﬀerent experimental conditions) from the pattern of mutations in a sample.

Our coalescent provides insight into the empirical work of France (2018), France, Ridenhour & Forney (2018) and France et al. (2019) that tracked the accumulation of mutations in several slow-growth settings in biofilms and chemostats. In particular, they observed the accumulation of mutations in *E. coli* forward in time for slow-growth (24 h doubling time) and fast-growth (2 h doubling time) chemostats and found that the fast-growth cultures had more mutations when compared over the same amount of (real) time. However, when they were compared over the same number of generations, the slow-growth cultures had significantly more mutations due to the fact that replication-independent mutations had more time to accumulate over their longer growth period (*i.e*., generation time). The growth rates in our simulations were chosen to match the relative rates in these experiments. It is worth remarking that population sizes were held fixed in our model and in the experiments of France (2018). This means that when comparing fast and slow growth populations, the fast growth population has faster turnover (*i.e*., replacement) of individuals; growth and death rates are both faster. If we were to only increase the growth rate, but not the death rate, we would be comparing populations with diﬀerent population sizes. Kaj & Krone (2003) and Sjödin et al. (2005) showed how an increase in population size increases diversity in a sample due to a slowing of coalescence rate.

**Figure 7:**
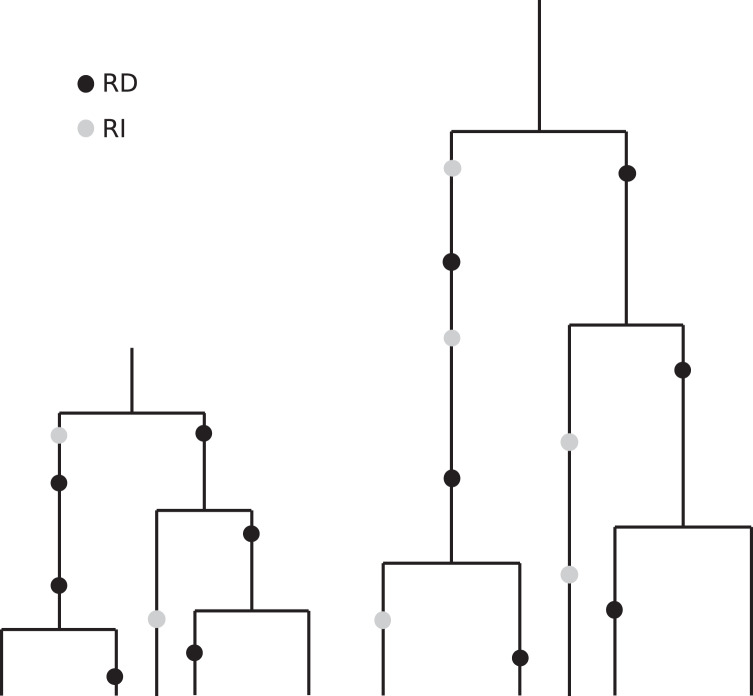
Illustration of the eﬀects of “real” time and generation time. This drawing indicates two coalescent trees having the same sample size and the same topology, with the one on the right corresponding to a slower replication rate. Although the number of generations is the same (and we have put the black RD mutations in the same relative positions for visual clarity), the number of time steps–real time–per generation is greater in the tree on the right. Since the grey RI mutations occur at a rate that is based on real time, we see more RI mutations in the tree on the right.

The coalescent, which is designed to account for the statistical patterns of SNPs in samples from a population, is generation-based and hence conforms more closely to comparisons that are made between cultures having the same number of generations. Specifically, coalescence events (that determine the branch lengths in the genealogy of a sample) occur according to generational time. These branches then accommodate replication-dependent mutation events (also generation based) and replication-independent mutations (occurring in real time). See Fig. 7 for an artist’s rendering of two coalescent trees that illustrate how slower replication rate leads to more RI mutations. The patterns of diversity generated by the coalescent with replication-independent mutations are in broad agreement with the empirical results from France (2018) and provide insight into the underlying mechanisms.

Our coalescent results for the case of constant, but reduced, growth rate show that the expected number of RI mutations in a sample will be proportional to *ν*/*c*. So even a very small RI mutation rate, *ν*, will significantly influence the diversity of a sample provided that the replication rate, *c*, is small enough. In environmental samples, for example, *c* can be extremely small. Using our unbiased estimator of *ν*, it will now be possible to quantify in the lab actual RI mutation rates for various bacterial species.

Blath et al. (2015) studied a related seed bank model and coalescent that focuses on dormancy instead of slow growth. Their simulations were limited to the case of no replication-independent mutations (*ν* = 0), though their model included the possibility of *ν* > 0. Unlike our model, which produces negative values of Tajima’s D and Fu and Li’s D when replication-independent mutations occur at positive rates, theirs yielded positive values of Tajima’s D and Fu and Li’s D even in the absence of replication-independent mutations. In their model, the dormant cells are sequestered (as would occur with spatial structure) and cells migrate slowly between this dormant group and the actively growing group; it is this spatial segregation that produces positive D statistics. As such, their model appears to be especially appropriate for the interesting case of biofilm growth. Our model is appropriate for well-mixed cultures. The fact that the two models make diﬀerent predictions about SNP data opens the possibility of using SNP data to infer the historical level of spatial structure or mixing in an environmental microbial population.

There is no selection in our model or in the Blath seed bank model. France (2018), France, Ridenhour & Forney (2018) and France et al. (2019) have demonstrated increased mutations in slow growth chemostat cultures, which are presumably neutral, and in biofilms, where diﬀerences in growth rate are depth dependent. Some of these mutations turned out to provide resistance to antibiotics—a selective pressure that was not present when the mutations were generated. This suggests that even a neutral coalescent with replication-independent mutations can inform the evolutionary dynamics of bacterial populations that eventually undergo selective pressure—such as through the use of antibiotics.

## Supplemental Information

10.7717/peerj.12926/supp-1 Supplemental Information 1 Python Code.

## Additional Information and Declarations

### Data Availability

The following information was supplied regarding data availability: The Python code for the model is available in the Supplemental File.
